# National Health and Modern Problems

**Published:** 1920-08-07

**Authors:** 


*To the Editor of* The Hospital.

### National Health and Modern Problems.

*To the Editor of* The Hospital.

Sir,—In writing a concluding letter, I would like to say that I have lived much of my own life in the "back-to-back" house. I have also seen these poor consumptive creatures dragging through dreary days, pale, wasted, and listless, in hospital. May I quote as follows from the Report of Ministry of National Service :—

"It is not good national hygienic economy to aim at immense commercial and industrial success if by so doing you produce a race of seniles at forty."

I quote the following from the current *Spectator*, reviewing "The Industrial Clinic": ". . . the reader must indeed be hard of heart who, studying the manual, does not feel increased sympathy with the worker." "It is indeed surprising to learn how many trades are associated with processes dangerous to health." "And it may be truly said that in some industries men have been and are being worked to death."

Who, reading these things, will not pause to think—more, to resolve?—Yours, etc., "Contemplator."

July 31, 1920.

